# Clinical use of molecular methods for *Trypanosoma cruzi* infection in endemic and non-endemic countries: Benefits, limitations and challenges

**DOI:** 10.3389/fpara.2023.1241154

**Published:** 2023-11-21

**Authors:** Maria-Jesus Pinazo, Colin J. Forsyth, Constanza Lopez-Albizu, Margarita María Catalina Bisio, Adriana González-Martínez, Laura Bohorquez, Jimy Pinto, Israel Molina, Andrea Marchiol, Rafael Herazo, Irene Losada Galván, Tayná Marques, Fabiana Barreira, Juan Carlos Villar, Yanina Sguassero, Maria Soledad Santini, Jaime Altcheh, Belkisyolé Alarcón de Noya, Sergio Sosa-Estani

**Affiliations:** ^1^ Drugs for Neglected Diseases initiative, Latin America, Rio de Janeiro, Brazil; ^2^ Red Nuevas Herramientas para el Diagnóstico y la Evaluación del Paciente con Enfermedad de Chagas (New Tools for the Diagnosis and Evaluation of Chagas Disease Network, NHEPACHA), Barcelona, Spain; ^3^ Centro de Investigación Biomédica en Red de Enfermedades Infecciosas, Instituto de Salud Carlos III (CIBERINFEC, ISCIII), Madrid, Spain; ^4^ Departamento de Investigación, Instituto Nacional de Parasitología (INP) “Dr. Mario Fatala Chaben”, Administración Nacional de Laboratorios e Institutos de Salud (ANLIS) “Dr. Carlos G. Malbrán”, Ministerio de Salud de la Nación, Buenos Aires, Argentina; ^5^ Consejo Nacional de Investigaciones Científicas y Técnicas (CONICET), Ministerio de Ciencia, Tecnología e Innovación de la Nación, Buenos Aires, Argentina; ^6^ Departamento de Investigación, Salvando Latidos AC, Guadalajara, Mexico; ^7^ Departamento de Investigación, Instituto Cardiovascular de Mínima Invasión, Guadalajara, Mexico; ^8^ Foundation for Innovative Diagnostics (FIND), Bogotá, Colombia; ^9^ Fundación Fundacion Ciencia y Estudios Aplicados para el Desarrollo en Salud y Medio Ambiente (CEADES), Cochabamba, Bolivia; ^10^ Fundação Oswaldo Cruz, Belo Horizonte, Brazil; ^11^ Barcelona Institute for Global Health (ISGlobal), Hospital Clínic - University of Barcelona, Barcelona, Spain; ^12^ Hospital Universitario 12 de Octubre, Madrid, Spain; ^13^ Fundacion Cardioinfantil, Bogotá, Colombia; ^14^ Facultad de Ciencias de la Salud, Universidad Autónoma de Bucaramanga, Bucaramanga, Colombia; ^15^ Instituto Nacional de Parasitología, Fatala Chaben-ANLIS, Buenos Aires, Argentina; ^16^ Dirección, Instituto Nacional de Parasitología (INP) “Dr. Mario Fatala Chaben”, Administración Nacional de Laboratorios e Institutos de Salud (ANLIS) “Dr. Carlos G. Malbrán”, Ministerio de Salud de la Nación, Buenos Aires, Argentina; ^17^ Servicio de Parasitologia y Chagas, Instituto Multidisciplinario en Investigaciones Pediatricas (IMIPP) CONICET- Hospital de Niños “Dr Ricardo Gutierrez”, Buenos Aires, Argentina; ^18^ Instituto de Medicina Tropical, Facultad de Medicina, Universidad Central de Venezuela, Caracas, Venezuela; ^19^ Centro de Investigaciones Epidemiológicas y Salud Pública (CIESP- CONICET), Buenos Aires, Argentina

**Keywords:** Chagas disease, *Trypanosoma cruzi*, polymerase chain reaction, loop-mediated isothermal amplification, laboratory diagnosis, treatment follow-up

## Abstract

*Trypanosoma cruzi* infection is diagnosed by parasitological, molecular, and serological tests. Molecular methods based on DNA amplification provide a more sensitive alternative to classical parasitological techniques for detecting evidence of *T. cruzi* parasitemia, and are the preferred tests for congenital and oral transmission cases and parasite reactivation in chronically infected immunosuppressed individuals. In newborns at risk of vertical transmission, simplified diagnostic algorithms that provide timely results can reduce the high follow-up losses observed with current algorithms. Molecular methods have also proved useful for monitoring *T. cruzi* infection in solid organ transplantation recipients, regardless of host immune status, allowing parasite detection even before symptom manifestation. Furthermore, in the absence of other biomarkers and a practical test of cure, and given the limitations of serological methods, recent clinical guidelines have included polymerase chain reaction (PCR) to detect therapeutic failure after antiparasitic treatment in chronically infected adults. Increasing evidence supports the use of molecular tests in a clinical context, given the improved sensitivity and specificity of current assays – characteristics which largely depend on epidemiological factors and genetic and antigenic variability among *T. cruzi* strains. Further development and registration of commercial PCR kits will improve the use of molecular tests. We discuss the attributes of PCR and other molecular tests for clinical management in people with *T. cruzi* infection.

## 1 Introduction

Diagnostic testing of *Trypanosoma cruzi* infection is at the heart of quality and comprehensive healthcare for people with Chagas disease, the majority of whom live in Latin America. However, over 90% of people currently with the infection lack access to tests, and less than 1% receive etiological treatment (Chaves et al., 2017). According to WHO, achieving elimination of Chagas disease (CD) as a public health problem by 2030 requires a radical simplification of current testing pathways to improve access and shorten time to diagnosis, and validation of the effectiveness of point-of-care diagnostics for infants and adults (World Health Organization, 2020).

The COVID-19 pandemic highlighted major inequities in access to testing. Only about 35% of COVID-19 tests administered worldwide have been used in low- and middle-income countries (LMICs), where most of the global population lives (Foundation for Innovative New Diagnostics, 2020). Furthermore, many test products for neglected tropical diseases, including CD, were discontinued during the pandemic, and global constraints on logistics severely impacted production of other diagnostics (World Health Organization, 2022).

*T. cruzi* infection presents in two phases: acute and chronic. Generally asymptomatic, the acute phase exhibits high parasite loads in peripheral blood, which decline to low or undetectable levels in the chronic phase. Therefore, direct diagnosis using parasitological tests should be carried out during the acute phase or at infection reactivation (Lopez-Albizu et al., 2023).

Microscopic detection tests with a concentration step have proven useful in reference centers (Feilij et al., 1983; Bisio et al., 2021). Requiring a centrifuge, a microscope, and, once implemented, specific training, they can be applied in settings with limited economic resources. However, their sensitivity and specificity have mostly been evaluated in referral centers, while low performance has been described elsewhere (Freilij and Altcheh, 1995; De Rissio et al., 2010; Messenger et al., 2017; Vera-Ku et al., 2019; Bisio et al., 2021).

Due to their high sensitivity, molecular nucleic acid amplification tests were evaluated for diagnosis and treatment follow-up of *T. cruzi* infection after the advent of PCR in 1991 (Avila et al., 1991; Britto et al., 1995; Russomando et al., 1998). These first assays (currently called endpoint PCR) were mostly used in research. However, their use later extended to clinical diagnosis (Schijman et al., 2011; Duffy et al., 2013), and subsequently other methods (isothermal tests) have been reported for *T. cruzi* detection (Thekisoe et al., 2007; Besuschio et al., 2017; Rivero et al., 2017).

For instance, qPCR has several advantages: it is amenable to automation, samples can be preserved for processing, and laboratory routines and internal and external quality control programs are required (Schijman, 2018; Bisio et al., 2021; Lopez-Albizu et al., 2023). However, supplies for molecular amplification tests are more expensive than those for microscopic tests. In areas without preexisting capacity, samples must be sent to higher complexity laboratories, whereas microscopic test results can be obtained in less than one hour after sample collection.

In recent studies using artificial samples containing cultured parasites, the limit of detection of qPCR has been described between 0.7 and 2.0 parasite equivalents per ml (Avila et al., 1991; Thekisoe et al., 2007). PCR has been proposed for early detection of parasite presence in acute vectorial/oral infection, vertical transmission, post-transplantation monitoring of seronegative recipients, and reactivated CD in immunosuppressed chronic patients (de Freitas et al., 2011; Velázquez et al., 2014; Noya et al., 2015; Messenger et al., 2017; Bisio et al., 2021). Moreover, qPCR has proven useful in early detection of therapeutic failure (Britto, 2009). Although PAHO has not included qPCR tests in recommendations for diagnosis of *T. cruzi* infection, they are used in some countries as an alternative to direct parasitological tests during the acute phase, reactivation, or vertical transmission (Ministerio de Salud de Panamá, 2012; Ministerio de Salud del Gobierno de Chile, 2017; Ministerio de Salud de la Nacion, 2022). Furthermore, the clinical use of a commercial loop-mediated isothermal amplification (LAMP) test is currently being evaluated in endemic regions.

qPCR methods that amplify nuclear satellite DNA (SatDNA) or kinetoplastid DNA (kDNA) sequences, which are present in multiple copies, are recommended for detecting *T. cruzi* in clinical samples (Schijman et al., 2011; Vasoo and Pritt, 2013). Two in-house qPCR tests targeting SatDNA and kDNA sequences have been analytically validated for quantification of *T. cruzi* in blood samples (Duffy et al., 2013; Ramírez et al., 2015; Cura et al., 2017). Recently, at least eight commercial qPCR tests for detecting *T. cruzi* infection have been developed, representing an important advance in the standardization of these techniques (Lopez-Albizu et al., 2023). The current availability of methods, and the capacity to conduct molecular detection of *T. Cruzi* with high sensitivity and specificity in clinical laboratories, may improve the diagnosis of CD.

It is important to note that *T. cruzi* is a heterogeneous parasite with high genetic and phenotypic diversity (Zingales et al., 2012). Currently, it comprises seven discrete typing units (DTUs), TcI-TcVI, and Tcbat. Although all DTUs can cause CD, they may exhibit different histotrophism, leading to diverse clinical forms and degrees of severity (Macedo et al., 2004). This genetic diversity has been related to geographical distribution, pathogenesis, clinical features, and response to serological tests and therapy (Zingales, 2018). Selecting the appropriate primers and target DNA sequences is important to ensure sensitive and specific detection in different geographic areas with different DTUs and variants.

Efforts have been made to assess the correlation between genotypic variability and phenotypic expression. Variances in replication, tissue tropism, or pathogenicity have been observed in experimental models. However, to date, clinical distinctions have solely emerged from observational studies that have not convincingly established a direct association between parasite genetic diversity and clinical manifestations (Vela et al., 2021).

## 2 Clinical use of molecular diagnostic techniques in acute *Trypanosoma cruzi* infection management

### 2.1 Molecular diagnosis tests in acute congenital infection

Considering PCR’s high sensitivity and specificity in patients with detectable parasitemia, compared with parasitological tests (Schijman et al., 2003) (Irurtia et al., 2021), it is essential for the diagnosis of patients in early disease stages. In patients with recent infection, independent of the transmission route, the sensitivity of qPCR is greater than 95%. In recent years, qPCR has been implemented as a highly sensitive test for the diagnosis of congenital CD (Bisio et al., 2021), since its sensitivity is higher in symptomatic patients. This is also the case for direct parasitological methods. Some research points to a correlation between higher parasite load in mothers and risk of congenital transmission (Bua et al., 2012). In asymptomatic infant patients, high parasite loads have been observed in infants <1 month old (Duffy et al., 2009; Benatar et al., 2021). Diagnosis at this age poses challenges, since serological methods are not useful in the first months of life, due to the passive passage of maternal antibodies. Current follow-up schemes, with serological studies beyond nine months of age, show high attrition rates of more than 40% (De Rissio et al., 2010). However, qPCR in this group avoids prolonged follow-up and has shown adequate sensitivity as a marker of therapeutic response, with observation of intra-treatment negativization in acute CD.

### 2.2 Molecular diagnosis tests monitoring acute infection during outbreaks

In outbreaks of orally transmitted *T. cruzi*, the emergency situation necessitates timely pre-treatment diagnosis, confirmation by PCR, and treatment administration (Noya et al., 2023). Oral transmission of *T. cruzi* to humans has probably always existed but was first reported in 1967 during outbreaks in the Amazon basin of Brazil, and later in Colombia, Venezuela, and French Guiana (Ruiz-Guevara et al., 2015). The largest outbreaks occurred in Venezuela in 2007 and 2009, in schoolchildren, from traditional guayaba juice contaminated by infected triatomines (Alarcón de Noya et al., 2010; Alarcón de Noya et al., 2016). Follow-up of 106 patients treated with nifurtimox was performed with cultures, serology, and PCR (Díaz-Bello et al., 2021), suggesting a high rate of therapeutic failure. Lytic antibody determination persisted in 79% of individuals evaluated at seven years, correlating well with the finding that 69% of these patients were positive by PCR at 10 years after treatment. In follow-up of 12 of these patients with qPCR, parasitic load initially decreased after treatment, but subsequently increased in those patients with therapeutic failure (Díaz-Bello et al., 2021).

## 3 Molecular diagnostic tests in cases undergoing reactivation of a chronic infection

Detection of the *T. cruzi* parasite in adults with acute infection is challenging. Serological tests are used for detection of *T. cruzi* infection in the chronic phase (Pan American Health Organization, 2019); however, serological tests may sometimes give false negative results in individuals in the early stages of infection or who are immunosuppressed (Rangel-Gamboa et al., 2019). In such cases, parasitological tests are useful, but patients often present several weeks after infection when the parasitic load has decreased considerably, hampering direct parasite observation on peripheral blood smear, microhematocrit, or Strout test in chronically infected adults (Britto, 2022). In these cases, other parasitological tests could be used, such as culture, but these methods are complex and time-consuming, delaying treatment initiation (Zavala-Jaspe et al., 2009; Dias et al., 2013). When microscopic methods fail to detect parasitemia, molecular tests have proven valuable; they can detect small amounts of parasite genetic material in a clinical sample (Schijman, 2018).

qPCR is one of the most reliable and widely used molecular tests for adults with acute *T. cruzi* infection or reactivation of CD by immunosuppression, because this test allows quantification of parasitic load and can differentiate between parasite strains (Schijman, 2018; Besuschio et al., 2020).

Nonetheless, qPCR has some limitations. It has not been tested in all endemic regions and is expensive, requiring adequate laboratory infrastructure, so its availability can vary. Areas with greater endemicity often do not have the resources to access this test.

## 4 Clinical use of molecular techniques for assessing response to treatment for *Trypanosoma cruzi* infection

### 4.1 Adult population in the chronic phase

Several studies in which serology and PCR have been used as surrogate markers of parasitic persistence after treatment have demonstrated the utility of PCR techniques in treatment follow-up. IgG-specific antibodies used as markers of parasitological response decrease 10 to 20 years after treatment and are the gold-standard long-term endpoint for evaluation of cure (Sguassero et al., 2018). Among CD patients in Brazil, 54.8% had positive serological tests 10.9 years/person after treatment (Neves Pinto et al., 2020). The low sensitivity of parasitological tests in chronic patients, coupled with the persistence of IgG antibodies for long periods, means that simple molecular tests are needed to detect therapeutic failure early. For example, therapeutic efficacy was assessed by qPCR in 31 of 48 (64.6%) patients treated for chronic CD after 20 years of follow-up (Britto et al., 2001). In a study in Chile, PCR sensitivity in patients with confirmed *T. cruzi* infection was 82.4%; after a mean of 6.6 years post-treatment, all patients persisted with positive serology while 6.1% exhibited positive PCR (Vergara et al., 2019). The use of qPCR has significantly improved assessment of response to treatment (Parrado et al., 2019) and has been used systematically in several clinical trials that have been accepted by regulatory agencies for clinical development (Ciapponi et al., 2023).

### 4.2 Pediatric population in the chronic phase

In children with chronic *T. cruzi* infection under 15 years of age (recent chronic infantile CD), the sensitivity of molecular techniques has exceeded 50% (González et al., 2022; Altcheh et al., 2023). The advantage of molecular methods as markers of therapeutic efficacy lies in their high sensitivity compared to other biomarkers. However, sensitivity decreases as time passes after infection (Vergara et al., 2019; Altcheh et al., 2021). Another advantage is the early detection of therapeutic failures, especially in children with chronic infection, facilitating decision-making for treating this population with the alternative therapeutic option (those treated with benznidazole (BNZ) are offered nifurtimox as second line and vice versa) (Moscatelli et al., 2019).

In a multicenter clinical study (Argentina, Bolivia, Colombia) that included patients aged 0 to 18 years, qPCR showed an overall sensitivity of 52% with a single sample. However, in infants under two years of age, the sensitivity was 95%. During follow-up, those qPCR positive at diagnosis became persistently negative, and > 90% were negative at four years of follow-up (Altcheh et al., 2023).

Few clinical studies have correlated genetic diversity with treatment response. There is some evidence from a non-comparative observational study conducted in Latin America, which evaluated seroconversion following treatment with BNZ in 2,840 children and adolescents. This study used patient origin as a proxy for genetic diversity. Seroconversion rates were rather variable: 3.1% in Bolivia, 58% in Guatemala, and 87% in Honduras (Yun et al., 2009).

## 5 Other potential uses of molecular diagnostic techniques in clinical management of chronic *Trypanosoma cruzi* infection

Parasite detection in adults with chronic *T. cruzi* infection is another challenge in areas with high genetic variability of the parasite, due to both pathogen characteristics and host immune responses that might impact the performance of diagnostic tests. In these areas, commonly used serological tests to diagnose chronic infection may be limited (Guzmán-Gómez et al., 2015). One potential strategy is direct detection of parasites by performing molecular tests on biological samples, such as blood or tissue (Zingales, 2018). Importantly, direct parasite detection by molecular tests does not replace serological testing, which is fundamental for diagnosis and surveillance of CD Ramsey 2023 (Personal Comm). Currently, this use is mainly limited to research. Recent evidence and inclusion in guidelines, and extensive networks established for COVID-19, dengue, and influenza, could facilitate implementation of such tests in the health system.

The prevalence of clinical forms and the severity of CD varies according to geographical region (Zingales, 2018; Zingales and Bartholomeu, 2022). An estimated 30-40% of people infected with *T. cruzi* develop irreversible lesions in the heart, esophagus, or colon (Rassi et al., 2010). Thus far, there is no evidence to identify which individuals will develop heart, digestive, or mixed pathology, or why in some regions more serious conditions develop (Freilij and Altcheh, 1995; Vera-Ku et al., 2019). Therefore, detection of different *T. cruzi* DTUs through molecular tests could have significant clinical implications. DTU characterization is also useful for understanding the epidemiology and geographical distribution of *T. cruzi* genotypes, facilitating study of disease transmission to help develop more effective control strategies (Guhl, 2013; Izeta-Alberdi et al., 2016; Villanueva-Lizama et al., 2019). Moreover, high parasitic load and persistent chronic infection have been postulated as risk factors for development of CD organ involvement. If confirmed through further research, molecular tests may prove useful for assessing CD severity and monitoring treatment response and effectiveness of interventions (Pinto et al., 2022).

Another key topic is the lack of knowledge on antiparasitic drug resistance and its potential correlation with genetic diversity. Clinical observations indicate a possible link between susceptibility or resistance to benznidazole and specific *T. cruzi* lineages or strains. While non-clinical evidence of drug resistance exists, there is a lack of research establishing a direct connection between therapeutic failure, strain resistance, and parasite DTU (Vela et al., 2021).

Recent clinical trials have attempted to correlate treatment failure with selection of resistant strains under treatment pressure. However, the genetic variability of *T. cruzi* bloodstream populations during post-treatment follow-up did not differ from that observed during chronic infection in the absence of treatment, suggesting that there were no selection events contributing to resistant parasite populations (Ramírez et al., 2022). Conversely, data obtained from an outbreak of acute oral Chagas disease in Venezuela demonstrated variation in the subpopulation of parasites before and after treatment, suggesting selection of a benznidazole-resistant population due to the effect of the drug (Muñoz-Calderón et al., 2021). Although evidence is still limited and controversial, it seems plausible that response to the drug might differ depending on the DTU. Recent clinical trials aim to explore this genetic diversity in treatment response (Molina-Morant et al., 2020).

## 6 Challenges and opportunities of the use of molecular techniques to detect *Trypanosoma cruzi* infection

### 6.1 Operational limitations in the use of PCR

During the COVID-19 pandemic, qPCR capabilities were implemented in most countries; this may facilitate including PCR into the diagnostic algorithms for CD in countries that do not yet do so, as recommended by PAHO-WHO (Pan American Health Organization, 2019). The disadvantages of PCR are the technology needed for implementation and the cost of supplies and reagents, especially for health systems still reeling from the pandemic. Although rare, technical issues can generate false positives, mainly due to contamination during extraction of DNA from samples. In order to avoid this, laboratories should perform these procedures in a protocolized manner, with strict internal and external quality control.

Sample volume is an important consideration. Since the parasites are not homogeneously distributed in blood, the greater the volume of blood, the greater the probability of finding *T. cruzi*. In newborns, a blood volume of 0.5 to 1 ml is suggested. In older children with early chronic infection, larger volumes of 2 to 5 ml are required to increase sensitivity (Schijman et al., 2003; Parrado et al., 2019). Preservatives and sample stabilizers, such as EDTA-guanidine buffer, are needed to preserve blood samples that are referred for testing in centers with PCR equipment. However, in centers where the PCR equipment is available nearby, the use of this preservative is unnecessary, lowering implementation costs.

Commercial kits usually only provide reagents for DNA amplification, leaving laboratory personnel to decide which DNA extraction techniques to use; this can cause difficulties in laboratories with a low volume of samples to be analyzed. To confirm congenital infection in asymptomatic children, tests should be repeated with a new sample, in order to avoid the possibility of missing a case in this high-priority population.

### 6.2 Decentralization of the clinical use of molecular diagnostic techniques: challenges and opportunities in the use of loop-mediated isothermal amplification

LAMP testing of *T. cruzi* potentially offers rapid detection of target DNA sequences, with results available within an hour, facilitating rapid clinical decision making (Schijman, 2018). It does not require a thermal cycling device since it operates isothermally at 60–65°C, and shows remarkable performance in terms of sensitivity and stability. Importantly, it does not have strict requirements in terms of infrastructure and resources, such as a reliable power supply or a temperature-controlled environment. This makes the implementation and scalability of decentralized LAMP diagnostics possible in low-resource settings (Notomi et al., 2000; Dea-Ayuela et al., 2018).

However, implementation of LAMP testing for infectious diseases has posed a challenge for health systems. Although LAMP has a lower cost than other methods, such as PCR (John et al., 2021), a thorough assessment of health economic variables is required to facilitate implementation at the point-of-care. Compared with active malaria case-finding microscopy, LAMP is generally more expensive, but has a lower cost per infection identified, and, thus, is more cost-effective due to its higher performance. An analysis of the prevalence, distribution, and mitigation of implementation costs, together with investing in supplies and training of personnel, could support the use of LAMP in some scenarios to improve infection management (Zelman et al., 2018). Additionally, LAMP does not allow precise genetic typing of *T. cruzi* for more detailed epidemiological investigations.

### 6.3 New landscape for molecular diagnosis

One positive consequence of the COVID-19 pandemic was that public literacy about diagnostics increased, with billions of people around the world undergoing COVID-19 testing and discussing different types of tests and test performance. There is a new appreciation in the global health research community for the need to develop and implement better performing tests, such as molecular diagnostics to improve the management of COVID-19 and other infectious diseases, including CD.

Since political interest in diagnostics has also increased, along with increased capacity for gene sequencing in LMICs, there is an opportunity to move access to routine molecular diagnostics for diseases with a high burden in the region, such as CD, up the priority list for future health reforms and investment in health systems.

Importantly, decentralized tools usually become accessible to high-income countries more easily than to LMICs, as was the case for COVID-19 diagnostics during the pandemic. At the market level in Latin American countries, several issues hinder availability and affordability of priority diagnostics. However, barriers to decentralized CD diagnostics can be overcome at the market level, for example, by centralizing purchase and procurement processes in several endemic countries. This would support sustained demand and, therefore, create a more attractive market for manufacturers, with diversification in diagnostics manufacturing supporting countries in the Global South to become more self-sufficient.

## 7 Conclusion

Molecular testing of *T. cruzi* could speed up diagnosis and evaluation of therapeutic response if implemented as part of comprehensive patient care. The use of molecular techniques in adults is already accepted, thanks to current evidence in acute cases, including oral outbreaks and congenital transmission. For chronic infection, there is enough evidence to recommend molecular tests for cases of reactivation, or to monitor for reactivation in people suffering from immunosuppression. Molecular tests have also been proposed for the assessment of therapeutic response in patients with chronic CD. To develop a reliable laboratory tool for treatment follow-up, several difficulties need to be addressed, such as the low and intermittent number, and mixed types, of circulating parasites during the chronic phase of infection. Additionally, to assess CD severity, we need more knowledge about how parasitological load relates to organ involvement. The role of parasite DTU in determining disease severity and treatment response is also a critical consideration for future implementation of molecular tools.

Care of patients with an early diagnosis of *T. cruzi* infection should take place in primary care facilities. Although there are already established diagnostic algorithms and patient flows, access is often limited to higher levels of care that remain difficult to access for most patients who might require PCR for diagnosis and therapeutic failure. This situation could be improved by providing access for these vulnerable populations to approaches such as LAMP; however, these techniques are still in the experimental phase of evaluation.

## Data availability statement

The original contributions presented in the study are included in the article/supplementary material. Further inquiries can be directed to the corresponding author.

## Author contributions

M-JP, CF, YS and SS-E contributed to the conception and design of the manuscript. CF, AG-M, CL-A, LB. MP coordinated the writing group. M-JP, CF, CL-A, MB, AG-M, LB, JP, IM, AM, RH, ILG, JV, JA, MS and BA wrote sections of the manuscript. M-JP, SS-E and CF wrote the merged draft of the manuscript. All authors contributed to the article and approved the submitted version.
